# Supplementary material for: Impact of the veterinary feed directive on Ohio cattle operations
Source: PLoS One. 2021 Aug 9;16(8):e0255911. doi: 10.1371/journal.pone.0255911 (PMC8351939; doi:10.1371/journal.pone.0255911)
Supplement: S1 File — (DOCX) [file pone.0255911.s001.docx]

**On-Farm Interview Questions**

Assessing the Role of Cattle Production Systems and Landscape Characteristics in Antimicrobial Resistance Profiles

*IRB Exemption Study ID: 2018E0880*

**FARM ID: _________________**

**Interviewer Initials: __________**

**Date: __________ Time: _________**

**IRB INFORMED CONSENT**

*As you know, we take privacy issues very seriously on this project. To ensure that all participatants in our study know what is happening, we’ve created an informed consent form that outlines our procedures and other relevant information.*

*Before we start – did you have a chance to review the consent form that I sent you earlier?*

*IF YES: Do you have any questions? (and here is a print copy)*

*IF NO: Here is another copy*

The key issues I’d highlight:

- **This study is entirely voluntary** – you can refuse to answer any questions or withdraw from the study at any time without penalty
- **The information we collect will be kept confidential**; individually identifying information will be removed from all datasets.
- **The benefits are twofold** – we can share a confidential report with individual participants for their farm, and results will contribute to our collective understanding of the role of landscape factors that shape farmers’ risks of dealing with antibiotic resistant bacteria.
- **There are minimal risks**.

**FARM CHARACTERISTICS:** *To begin, I’d like to learn more about your operation. These first questions help us get a sense of how your (beef/dairy) operation is organized.*

***BEEF COW-CALF / BEEF FEEDLOT / DAIRY HEIFER or DAIRY BEEF OPERATIONS***

- - **How would you describe your operation?** *(cow-calf, heifer raising, stockers, feedlot/finish, etc.)*

*________________________________________________________________________*

*________________________________________________________________________*

*________________________________________________________________________*

*________________________________________________________________________*

- - **On average, how many cattle and calves do you have on the farm?** (beef cows, calves, heifers, steers, bulls, etc.)

TYPE Age Classes NUMBER

Mature COWS (have calved) _________

Young CALVES (under 6 months) _________

HEIFERS kept for breeding (6 mos to fresh) _________

STEERS and other beef (6 mos to sale) _________ *(note – heifers for beef go here)*

Other: ________________ ____________ _________

- - **Where are your cattle and calves located now?**

# CATTLE:_________________________________________________________________________________________________________________________________________________________________________

# HEIFERS/STEERS__________________________________________________________________________________________________________________________________________________________________

# CALVES:_________________________________________________________________________________________________________________________________________________________________________

- - **How would you describe your cattle housing?** *(Type of housing, note bedding)*

COWS:____________________________________________________________________________

# 🞎 Straw 🞎 Sand 🞎 Sawdust 🞎 Mattresses

CALVES:___________________________________________________________________________

# 🞎 Straw 🞎 Sand 🞎 Sawdust 🞎 Mattresses

OTHER:___________________________________________________________________________

# 🞎 Straw 🞎 Sand 🞎 Sawdust 🞎 Mattresses

- - **Do any of your cows regularly have access to pastures? ___ Yes ___ No**

# If yes, describe: ________________________________________________________

# _____________________________________________________________________________

# _____________________________________________________________________________

*_____________________________________________________________________________*

**SKIP TO PAGE 6**

***MILKING DAIRY HERDS***

- - **How big is your milking herd?** *_____* ***(Include milking and dry cows)***
  - **What would estimate is your average level of herd productivity** (either rolling herd average or lbs/cow/day)?

____________lbs/cow/day ___________ RHA

- - **What other types of cattle (heifers, calves, bulls, etc.) do you have on the farm?**

TYPE Age Classes NUMBER

Young CALVES (under 6 months) _________

HEIFERS kept for breeding (6 mos to fresh) _________

STEERS and other beef (6 mos to sale) _________ *(note – heifers for beef go here)*

Other: ________________ ____________ _________

_____________________ ____________ _________

- - **Where are your cattle housed or located right now?**

# MILK COWS: ___________________________________________________________________

# HEIFERS: ___________________________________________________________________

# CALVES: _____________________________________________________________________

OTHER:________________________________________________________________________

- - **What types of housing do you use for your dairy herd? What kinds of bedding do you use?**

MILKING HERD? Describe: ________________________________________________________

🞎 Tie stall or stanchion barn 🞎 Free stall barn 🞎 Other: ______________

Bedding: 🞎 Straw 🞎 Sand 🞎 Sawdust 🞎 Mattresses 🞎 Other: _____________

DRY COWS/HEIFERS? Describe: _________________________________________________

🞎 Not kept here 🞎 Free stall barn 🞎 Bedded pack/pen 🞎 Other: ______________

Bedding: 🞎 Straw 🞎 Sand 🞎 Sawdust 🞎 Mattresses 🞎 Other: _____________

YOUNG CALVES? Describe: _______________________________________________________

- 🞎 Indoor housing 🞎 Outdoor housing 🞎 Individual 🞎 Group

Bedding: 🞎 Straw 🞎 Sand 🞎 Sawdust 🞎 Mattresses 🞎 Other: _____________

OTHER? Describe: _______________________________________________________

- 🞎 Indoor housing 🞎 Outdoor housing 🞎 Individual 🞎 Group

Bedding: 🞎 Straw 🞎 Sand 🞎 Sawdust 🞎 Mattresses 🞎 Other: _____________

- - **Do any of your cows regularly have access to pastures? ___ Yes ___ No**

# If yes, describe: ________________________________________________________

# _____________________________________________________________________________

# _____________________________________________________________________________

# _____________________________________________________________________________

- - **(If they are ever out…) Do you rely on pastures for a significant percent of forage intake for your milking herd during grazing months?**

🞏 NO 🞏 YES

**BOTH DAIRY & BEEF OPERATIONS CONTINUE HERE**

**CATTLE MOVEMENT**

- - **Do you ever bring cattle onto this farm from other locations ?**
    - If yes, **what types & how many?**

_______________________________________________

_______________________________________________

- - - **How often**? ____________________________
    - **Where do these cattle typically come from?**
      - How far away? ________________ Within the county? __YES __NO
  - **When cattle or calves leave your farm – where do they usually go? How are they marketed?**

_________________________________________________________

_________________________________________________________

_________________________________________________________

_________________________________________________________

**OTHER LIVESTOCK**

- - **Do you have any other livestock on the farm?** (Horses, pigs, chickens, sheep, goats, etc.?)
    - If yes – what kinds & how many:

____________________________________ __________

____________________________________ __________

____________________________________ __________

____________________________________ __________

- - **Do you regularly bring any other livestock (non-cattle) onto the farm? How many?**

Hogs___ Poultry ___ Horses___ Sheep or Goats___

Other, specify _____________

- - **Do you have any farm pets** (dogs & cats)? ______________

**WATER SOURCES**

- - **What water sources are available for the cattle on your farm?**

# **In the barns / feedlots?**

- Waterers or Troughs in barn/buildings
- Waterers or Troughs in barnyard
- OTHER: _______________________________________________

# **In pastures?**

- Waterer or Troughs in pastures
- Natural streams or creeks
- Natural and/or Manmade ponds
- Seasonal pools (Vernal) – puddles in field?
- OTHER: _______________________________________________
  - **If you use troughs or waterers:**

# Is this water from a well, spring, or a city water system?

_________________________________________

**CROPS**

- - **How many total acres do you manage or operate as part of this farm? (include both owned & rented acres)**

**TOTAL SIZE OF OPERATION: ________**

- - - How much of that is in row crops (corn, soy, small grains) ________
    - How much of that is in hay? ________
    - How much (if any) is used primarily for grazing? ________
    - How much is in woods? ________
    - Any OTHER ACRES: _______________________________ ________

**CATTLE FEEDING PRACTICES**

- - **Do you use a nutritionist or feed consultant?** *___YES ___NO*
  - **Do you use a TMR (total mixed ration)?** *___YES ___NO*
  - **Do you usually bring in any of your cattle feed from off the farm?**

*___YES ___NO (if yes, describe:)*

___ forages ___ grains ___ mineral

Other: ________________________________________________________

- - **Is any of it produced locally?**

*___YES ___NO (if yes, describe:)*

*_____________________________________________________________________*

*_____________________________________________________________________*

**MANURE MANAGEMENT**

- - **How do you handle most of the manure from your cattle?** *(where stored, how handled, when spread)*

# _____________________________________________________________________________

# _____________________________________________________________________________

# _____________________________________________________________________________

- - **Do you usually spread your stored manure on any of your farm fields? _____Yes ____No**
  - If so, **which types of fields** do you try to put manure on? **__________________________________________________________________________________________________________________________________________**
  - **Do you usually put manure on pasture or hay fields?** _____Yes ____No
  - **On how roughly many acres did you spread manure on last year?** *(2018)* ________________
  - **Do you ever bring any manure or compost onto your farm from other farms** (to fertilize your fields)**?**

___ NO ___ YES 🡺 when last __ in last year __ in last 5 years __ before that

*Describe (source, type, where): ____________________________________________________* **________________________________________________________________________________________________________________________________________________________**

**RECENT FARM CHANGES:**

Over the past 10 years **has the scale or mix of farm enterprises on this farm changed significantly?** *(i.e.,farm type shift- shift from livestock to crop or from one type of livestock to another)?*

*___YES ___NO (if yes, describe:)*

**_____________________________________________________________________**

**_____________________________________________________________________**

**_____________________________________________________________________**

**FARM BOUNDARIES, NEIGHBORING PROPERTIES & LIVESTOCK OPERATIONS**

1. Because our focus in on capturing the landscape drivers of antimicrobial resistance, it can be helpful to get a sense of what is going on around your farm. To keep this managable, it might be good to start by thinking of the properties that border on parts of your current operation that regularly have livestock (housing or grazing). Before we came, we printed out *an aerial photo or satellite image (Zoomed in and Out) to guide our conversation – does this seem accurate?*
   - Looking at this aerial photo or image, **where on this map are cattle currently located?**

_______________________________________________________________

_______________________________________________________________

- - **What are the rough boundaries of the farm,** *focusing on areas around where livestock are located?*

_______________________________________________________________

_______________________________________________________________

- - **How are each of the main neighboring properties that border these areas currently being used?** (farming type, urban or developed, natural areas, mining/fracking)

_______________________________________________________________

_______________________________________________________________

- - **Roughly, how many livestock farms do you think are within a mile of your property?** *(include farms of any size i.e including neighbors with small numbers of livestock of any type)*

_________________

- - - **What types of livestock? Any of them commercial or large scale?**

___________________________________________________________

___________________________________________________________

- - **Where is the nearest hospital?**

**_______________________ ______ miles away**

- - **Where is the nearest sewage/ wastewater treatment facility?** *(okay to mark don’t know)*

**____________________ ______ miles away**

**HERD HEALTH:** Next, I want to ask some questions about how you manage your herd’s health.

1. **What would you say are the most challenging or pressing cattle health issues you have to deal with?** *(note if it is a disease that might have been treated w/ antibiotics so you can probe for recent instances below)*
2. ___________________________________________________________________________
3. ___________________________________________________________________________
4. ___________________________________________________________________________
5. ___________________________________________________________________________
6. ___________________________________________________________________________

**“Just to be sure we didn’t miss any – do you ever deal with…”** *Prompts to check for common things:*

*Mastitis, Respiratory diseases, Pinkeye, Scours, Johne’s, BVDV-Bovine Viral Diarrhea, Lameness/Footrot, hairy warts, parasites, blackleg disease, Breeding/reproduction or calving issues, metritis/post calving uterine infections*

**VACCINATION USE:**

1. **Do you vaccinate any of your cattle?** 🞎 YES 🞎 NO
2. **Is there a particular vaccination schedule you adhere to?** 🞎 YES 🞎 NO
3. **What vaccinations are routinely administered in your dairy or beef herd?**

CALVES:

________________________________________________________________________________________________________________________________________________________________________________

BRED COWS: ________________________________________________________________________________________________________________________________________________________________________________

HEIFERS:

________________________________________________________________________________________________________________________________________________________________________________

Other:

________________________________________________________________________________________________________________________________________________________________________________

**ANTIBIOTIC USE**

1. **Do you ever use antibiotics with your dairy or beef cattle?**

🞎 YES 🞎 NO 🡺 (if not – probe for reasons)______________________________

____________________________________________________

1. **In general – how would you describe your overall approach to using antibiotics in your herd?**

____________________________________________________________________________________________________________________________________________________________________________________________________________________________________________________________________________________________________________________________________

1. **Who usually administers antibiotics on your farm?** __________________________________
2. **Where do you usually purchase antibiotics for your farm?** __________________________________
3. **When were antibiotics last administered to any of your dairy or beef cattle?**
   - In last week 🞎 In last month 🞎 Last 3 months
   - More and 3 months ago (specify): ______________________________________________

**NEXT I’D LIKE TO REVIEW A FEW SPECIFIC EXAMPLES OF HOW YOU MADE DECISIONS ABOUT ANTIBIOTIC USE WITH YOUR BEEF OR DAIRY CATTLE**

Thinking of the most recent use of antibiotics, can you explain

- - **What was the situation that prompted use of antibiotics?**

*Type (beef vs dairy, cow vs. calf) and number of animals:*

*Type of illness, injury, disease:*

- - **What antibiotic(s) were used?**
  - **How did you decide whether to use antibiotics (and what to use)?**
  - **How long were the antibiotics used? (start/end dates)**
  - **How did they work?**

| **When was this, and how many animals were involved** *(each Instance plus recurrences)* | | | **Type of Illness/Infectious Disease** | **What antibiotics were used?** | **How did you decide whether to use antibiotics (and what to use)?** | **Treatment protocol** | **How did they work? Were they effective?** |
| --- | --- | --- | --- | --- | --- | --- | --- |
| DATE | # animals | Type of Animals | Metritis, Dry Cow Therapy,Mastitis, Respiratory illness, Diarrhea, Lameness, etc. | BP48 (penicillin G), Liquamycin (LA200), OxyTet, Terramycin, Terra-Vet Tylan, Dur-Pen, Agri-Mectin, Sustain, Bio-Mycin, Albon, Nuflor, Naxcel, Draxxin, Excede, Excenel, Advocin, etc. | Veterinary Consult? Personal diagnosis? Routine use? | Describe how often administered, for how long? (# shots given, spacing btw shots, switch to new antiobiotic, etc.) | Yes? If no – what did you do next? |
|  |  |  |  |  |  |  |  |
|  |  |  |  |  |  |  |  |
|  |  |  |  |  |  |  |  |
|  |  |  |  |  |  |  |  |

Notes: ____________________________________________________________________________________________________________________________________________________________________________________________________________________________________________________________________________________________________________________________________

1. **Do you usually separate animals receiving antibiotics from the herd?** ____Yes ____No
2. **Have you ever noticed problems with antibiotic effectiveness when treating your animals?**

____Yes ____No IF YES-- is this increasing over time? _______________________

1. **How concerned are you about the possibility that livestock diseases you need to treat will become resistant to antibiotics?**

 not at all concerned  a little concerned  somewhat concerned  very concerned

**VETERINARY FEED DIRECTIVE:**

In 2017, the US Food & Drug Administration (FDA) issued a ‘veterinary feed directive’ that imposed new rules for how medically important antibiotics can be administered to animals. Generally speaking, this rule requires more veterinary oversight (e.g., a prescription) whenever such antibiotics are fed to any food-animal species.

1. **How did this change affect your livestock operation? (What were the biggest challenges you faced?)**

___________________________________________________________________________________________________________________________________________________________________________________________________________________________________________________

**I now have a set of closed answer questions – for each one, I want you to indicate the extent to which the VFD changed any of the following things on your farm?** (answers range from decreased a lot to no change to increased a lot)

|  | Decreased a lot | Decreased a little | No change | Increased a little | Increased a lot |
| --- | --- | --- | --- | --- | --- |
| Use of Antibiotics: |  |  |  |  |  |
| Use of Vaccines: |  |  |  |  |  |
| Use of nutritional supplements in feed: |  |  |  |  |  |
| Animal Health: |  |  |  |  |  |
| Farm Profitability: |  |  |  |  |  |
| # of vet interactions |  |  |  |  |  |

1. **BOTTOM LINE: How difficult has it been for your operation to comply with the VFD?**

__________________________________________________________________________________________________________________________________________________________________

**WILDLIFE CONTACT**

In order to understand possible ways that resistant and non-resistant bacteria are circulated among farms, we are interested in your observations about the presence of wildlife on your farm.

**For each, can you let me know**

- **How often you see them on your farm** (Define the categories for participant)
- **Where you usually see them on your farm** (Derfine locations)

| **Wildlife Type** | **How often do you see them?** | | | | **Where do you see them?**  *(check all that apply; add notes if necessary)* | | | | |
| --- | --- | --- | --- | --- | --- | --- | --- | --- | --- |
|  | **Never** | **Rarely** (~1/year) | **Occasionally** (~monthly) | **Frequently**  (weekly to daily) | **Buildings** | **Cropland**  *(note if corn or hay)* | **Pasture** | **Forest/Natural areas** | **Water Source** *(note if cattle drinking water or just nearby pond)* |
| **White-tailed Deer** |  |  |  |  |  |  |  |  |  |
| **Squirrels/**  **Rabbits** |  |  |  |  |  |  |  |  |  |
| **Coyote** |  |  |  |  |  |  |  |  |  |
| **Fox** |  |  |  |  |  |  |  |  |  |
| **Bobcat** |  |  |  |  |  |  |  |  |  |
| **Turkeys** |  |  |  |  |  |  |  |  |  |
| **Geese/Ducks** |  |  |  |  |  |  |  |  |  |
| **Rodents (mice, rats, voles)** |  |  |  |  |  |  |  |  |  |
| **Raccoons, skunks, opossum, groundhogs** |  |  |  |  |  |  |  |  |  |
| **Pigeons, starlings, sparrows, swallows** |  |  |  |  |  |  |  |  |  |
| **Bats** |  |  |  |  |  |  |  |  |  |
| **OTHER:** |  |  |  |  |  |  |  |  |  |

***Notes about where we might go find deer:***

_______________________________________________________________________________________

**Do you actively try to reduce or eliminate wildlife on your farm? __No __ Yes: ________________________**

**Do you do anything to attract birds and wildlife on your farm? __No __ Yes: __________________________**
