# Supplementary material for: Impact of the veterinary feed directive on Ohio cattle operations
Source: PLoS One. 2021 Aug 9;16(8):e0255911. doi: 10.1371/journal.pone.0255911 (PMC8351939; doi:10.1371/journal.pone.0255911)
Supplement: S2 File — (DOCX) [file pone.0255911.s002.docx]

## Phase 2: Cattle Farm Interview Questions

**FARM INTERVIEW**

**Assessing the Impact of the Veterinary Feed Directive**

**on Ohio Cattle Operations**

In 2017, the US Food & Drug Administration (FDA) issued a Veterinary Feed Directive’ (VFD) that imposed new rules for how medically important antibiotics can be administered to animals. Generally speaking, this rule requires more veterinary oversight whenever such antibiotics are fed to any food-animal species.

The purpose of my study is to gauge the impact of these new rules on producers, and to get a sense how things unfolded on the ground. We will share your experiences - positive, negative, or neutral – with decision makers and researchers working to find practical approaches to managing herd health in the beef and dairy industry.

*We take privacy issues very seriously on this project and it is important to us that you understand your rights and role in this study.*

*Please know:*

- **This study is entirely voluntary** – you can refuse to answer any questions or withdraw from the study at any time without penalty
- **The information we collect will be kept confidential –** individually identifying information will be removed from all datasets.
- **The benefits** – Results will contribute to our collective understanding of livestock health issues and the impact of new regulations on farm operations
- **There are minimal risks –** All materials have been approved by The Ohio State University (IRB # 2019E0899)

**I. Description of Farm**

1. How would you describe your cattle operation?

- e.g., *Dairy, Cow/calf, Heifer raising, Stocker, Finishing/Feedlot, Seedstock / Registered Breeder, Other*

1. How many total acres are in your farm operation? ___________
2. How many of each of the following type of cattle do you currently have on the farm?
   1. Beef or milk cows:
   2. Other adult cattle (over 6 months; steers, heifers, bulls):
   3. Calves under 6 months:

___________

4. Do your adult cattle have access to pastures? – *pick the answer that best applies*

- No, never or rarely
- Yes, seasonally
- Yes, most or all of the time

5. Do you use a TMR (total mixed ration) machine? (Yes or No)

1. How often do you bring new cattle into your operation from other farms? – *pick the answer that best applies (*Never or rarely; Occasionally; Frequently)
2. Do you quarantine new animals upon arrival? (Always / Sometimes / Never / NA)
3. Do you have any other types of livestock on this farm? (e.g. hogs/poultry/sheep)

**II. Herd Health Management**

1. What would you say are the most challenging or pressing cattle health issues you have to deal with?
2. Do you regularly vaccinate any of your cattle? (Yes or No)
3. What is your overall approach to antibiotic use?
4. Over the last 12 months, have you used any antibiotics in your dairy or beef herd?
   1. Yes/No
   2. If yes- when were antibiotics last administered to any of your dairy or beef cattle?
   3. For what kinds of conditions did you use antibiotics over the last year?
5. Have you ever noticed problems with antibiotic effectiveness when treating your animals?
6. How often does a vet typically visit your farm in person? – *pick the answer that best applies (*At least once a month; Less than once/month but more than once/year; Less than once/year)

**III. VFD**

*As you probably know, in 2017, the US Food & Drug Administration (FDA) issued a ‘Veterinary Feed Directive’ (VFD) that issued new rules for how medically important antibiotics can be administered to animals. Generally speaking, this rule requires more veterinary oversight (e.g., a prescription) whenever such antibiotics are fed to any food-animal species.*

1. How did this change affect your livestock operation?
2. What were your feelings when the VFD first came out?
   1. Did you feel it was needed?
   2. Did you feel it was going to be effective or useful?
3. How do you feel about the VFD now?
4. Before the VFD, how often did you mix antibiotics with cattle feed or water on your farm?
5. Routinely B. Occasionally C. Rarely D. never

If A, B, or C

- please briefly describe the kinds of situations where you used antibiotics in feed or water for livestock:
- Since the VFD – have you been able to continue using antibiotics in your livestock food or water?

1. Please indicated how the VFD has affected your operation in each of the following ways:

5 = Increased a lot

4 = Increased a little

3 = no change

2 = Decreased a little

1 = Decreased a lot

|  | **1** | **2** | **3** | **4** | **5** |
| --- | --- | --- | --- | --- | --- |
| Use of antibiotics in food or water |  |  |  |  |  |
| Use of antibiotics in general |  |  |  |  |  |
| Use of vaccines |  |  |  |  |  |
| Use of nutritional supplements in feed |  |  |  |  |  |
| Number of vet interactions |  |  |  |  |  |
| Livestock Health |  |  |  |  |  |
| Farm profitability of Farm |  |  |  |  |  |
| Amount of paperwork |  |  |  |  |  |
| Other (specify) |  |  |  |  |  |

1. To summarize: What was the greatest challenge for your operation in complying with the VFD?
2. What strategies or responses did you use to deal with this challenge?
3. Bottom line: How difficult has it been for your operation to comply with the VFD?

**IV. Information about you**

1. How long have you been farming?
2. Please select your age range:

- 18 – 25
- 26 – 35
- 36 – 45
- 56 – 65
- 66 – 75
- > 75

1. What is your highest level of formal education?

___high school ___ some college ____ 4 year college degree

___graduate degree _____ other (describe)

*Finally – I have a few questions about your level of concern about the issues surrounding antibiotic resistance.*How concerned are you about the possibility that livestock diseases you need to treat will become resistant to antibiotics?

 not at all concerned  a little concerned  somewhat concerned  very concerned

1. How concerned are you that antibiotics used in cattle are becoming less effective?

 not at all concerned  a little concerned  somewhat concerned  very concerned

1. How concerned are you that human diseases are becoming more resistant to antibiotics?

 not at all concerned  a little concerned  somewhat concerned  very concerned

1. Finally, please indicate your extent of agreement or disagreement with each of the following statements:

- “The use or overuse of antibiotics in livestock production is a contributing factor to antibiotic resistance found in livestock” (SD/D/N/A/SA)
- “The use or overuse of antibiotics in livestock production is a contributing factor to antibiotic resistance found in humans” (SD/D/N/A/SA)

1. What other thoughts or information would you like to share with researchers and policy makers on this topic?
2. Do you have any other comments or questions for us?

THANK YOU for your time and feedback!
