# Supplementary material for: Impact of the veterinary feed directive on Ohio cattle operations
Source: PLoS One. 2021 Aug 9;16(8):e0255911. doi: 10.1371/journal.pone.0255911 (PMC8351939; doi:10.1371/journal.pone.0255911)
Supplement: S3 File — (DOCX) [file pone.0255911.s003.docx]

Phase 2: Veterinarian Interview Questions

**VETERINARIAN INTERVIEW**

**Assessing the Impact of the Veterinary Feed Directive**

**on Ohio Cattle Operations**

In 2017, the US Food & Drug Administration (FDA) issued a Veterinary Feed Directive’ (VFD) that imposed new rules for how medically important antibiotics can be administered to animals. Generally speaking, this rule requires more veterinary oversight whenever such antibiotics are fed to any food-animal species.

The purpose of my study is to gauge the impact of these new rules on producers, and to get a sense how things unfolded on the ground. We will share your experiences - positive, negative, or neutral – with decision makers and researchers working to find practical approaches to managing herd health in the beef and dairy industry.

*We take privacy issues very seriously on this project and it is important to us that you understand your rights and role in this study.*

*Please know:*

- **This study is entirely voluntary** – you can refuse to answer any questions or withdraw from the study at any time without penalty
- **The information we collect will be kept confidential –** individually identifying information will be removed from all datasets.
- **The benefits** – Results will contribute to our collective understanding of livestock health issues and the impact of new regulations on farm operations
- **There are minimal risks –** All materials have been approved by The Ohio State University (IRB # 2019E0899)

**Interview ID: Date:**

1. How would you describe your veterinary practice? What type of animals do you work with?
2. How long have you been working as a large animal vet?
3. What share of your current clients are beef or dairy farmers? Percentage? # of VCPRs?
4. Do you also work with any poultry or hog farmers? Percentage? # of VCPRs?
5. In your experience, what are the most common reasons that antibiotics are used by beef producers you work with?
6. How about for dairy farms - what are the main reasons antibiotics are used on dairy operations??
7. How about for hog and poultry farms?
8. What is your personal/professional opinion of how AB should be used in food animals?
9. How recently have you prescribed AB? Why?
10. Have you noticed any change in AB effectiveness?
11. How familiar were you with the FDA Veterinary Feed Directive when it was issued in 2017?
    1. Did you participate in any discussions about what it should include when it was being drafted?
    2. What kind of training or info did you get when it was issued?
12. What were your feelings when the VFD first came out?
    1. Did you feel it was needed?
    2. Did you feel it was going to be effective or useful?
13. How do you feel about the VFD now?
14. How has the use of antibiotics on farms you work with changed since the implementation of the VFD?
15. Before the VFD, did you regularly recommend the administration of antibiotics through livestock feed or water?
16. How common was that practice among your clients before the VFD?
17. Did the VFD change your approach or recommendations with respect to administering antibiotics through livestock feed and water?
18. Overall, how did the implementation of the VFD affect your veterinary practice?
19. For each of the following, please indicate how much each of the following things have changed as a result of the VFD:
    *( decreased a lot; decreased a little; no change; increased a little; increased a lot)*

- # of Livestock clients wit VCPR
- Amount of AB you prescribe to be administered in feed or water
- Amount of AB you have prescribed for preventative/prophylactic reasons
- Amount of AB you have prescribed (total)
- Number of farm visits you make with clients
- Farm operator use of vaccines
- Farm operator use of feed supplements
- Farm operator use of fed antibiotics (in feed or water)
- Farm operator use of all antibiotics
- Overall health of livestock
- Herd or flock size
- Profitability of farms you work with
- Amount of paperwork

1. To summarize: What has been the greatest challenge for you and your veterinary practice in complying with the VFD?
2. What has been the greatest challenge for your farmer clients in complying with the VFD?
3. What kinds of livestock farms were most impacted by the VFD?
4. How have they been dealing with this challenge?
5. **Bottom line**: How difficult has it been for you and your clients to comply with the VFD?
   Does it vary with type of livestock?

*Finally – I have a few questions about your level of concern about the issues surrounding antibiotic resistance.*

1. How concerned are you about the possibility that livestock diseases you need to treat will become resistant to antibiotics? *(at all concerned; a little concerned; somewhat concerned; very concerned)*
2. How concerned are you that antibiotics used in cattle are becoming less effective? *(at all concerned; a little concerned; somewhat concerned; very concerned)*
3. How concerned are you that human diseases are becoming more resistant to antibiotics? *(at all concerned; a little concerned; somewhat concerned; very concerned)*
4. Finally, please indicate your extent of agreement or disagreement with each of the following statements:

- “The use or overuse of AB in livestock production is a contributing factor to ABR found in livestock ” *(SD/D/N/A/SA)*
- “The use or overuse of AB in livestock production is a contributing factor to ABR found in humans” *(SD/D/N/A/SA)*

1. What other thoughts or information would you like to share with researchers and policy makers on this topic?
2. Do you have any other comments or questions for us?

Thank you for your time and feedback!
